# Supplementary material for: Chromothripsis during telomere crisis is independent of NHEJ, and consistent with a replicative origin
Source: Genome Res. 2019 May;29(5):737–49. doi: 10.1101/gr.240705.118 (PMC6499312; doi:10.1101/gr.240705.118)
Supplement: Supplemental Material [file supp_gr.240705.118_Supplemental_file_1.zip › contigs/annotated_contigs/DB51/contig.2.DB51_length_736_mean_cov_8.5597826087.docx]

**DB51_length_736_mean_cov_8.5597826087**

```
TACTCATCAATAAAAATTCTCACTTTCAGTACACAGACTTCTCCTTCATTTCTCAGTTTTTACCTTCATGCCTCATCGTTTCCATTCTT
 >chr3:53499804-53500192 - E=1e-221
GAATTACAGTCCTCTGATTGGGGGATGGTTCCCACTTCTTCAGAGACTACACCCTTTGACTCTTTCATGGGTGGCAGGGGAAGCAGTTA

AAAAGGAGCTAAACTGATGCACACAATGACATAGATGAGCCTCACAGACACTGTGTTGAGCCATAAGACACAAAACAGTACATTTGTAT

GATTTCATTTATATGAATATGTTGACAAACGACTAAGTTATGGTGAAAAAAACACATAATAGAGGATGCCTCTGGAGGGGATGAGAAGG

AATGTGAAGAAACTTTCTGGGGTGATGGAA|AT|AAAAAAGGAATGAATTAATGGCATTTGCAGCAACCTGGATGAGACTGGAGACTAT
                               >chr3:53497866-53498216 - E=9e-199
TAGAAGTAACTCAGGAATGGAAAACCAAACATCATATGTTCTCACTCGTAAGTGAGAGCTAAGCTTATGAGGATGCAAAGGCATAAGAA

TGACACAATGGACTCTGGGGACTCAACAGGAAAGCGTGGGAAAGTGGTGAGGGATAAAAGACTACAAATTGGGTGCAGTGTATACTGCT

TGGGTGATGGGTGCACCAAAATCTCACAAATCACCACTAAAGAACATACTCATGTAATGAAACACCACTTGTTCCCCACTAACCTATGG

AAATAAATTTTTTTAAATAGTGATAA
```
